# Novel Antibacterial Properties of the Human Dental Pulp Multipotent Mesenchymal Stromal Cell Secretome

**DOI:** 10.1016/j.ajpath.2022.02.005

**Published:** 2022-03-23

**Authors:** Harriet Ravenscroft, Ikhlas El Karim, Anna D. Krasnodembskaya, Brendan Gilmore, Imad About, Fionnuala T. Lundy

**Affiliations:** ∗ Wellcome-Wolfson Institute for Experimental Medicine, School of Medicine, Dentistry and Biomedical Sciences, Queen's University Belfast, Belfast, United Kingdom; † School of Pharmacy, Queen's University Belfast, Belfast, United Kingdom; ‡ Faculté des Sciences Médicales et Paramédicales, Ecole de Médecine Dentaire, Centre National de la Recherche Scientifique, Institut des Sciences du Mouvement, Aix Marseille University, Marseille, France

## Abstract

It is well recognized that clearance of bacterial infection within the dental pulp precedes pulpal regeneration. However, although the regenerative potential of the human dental pulp has been investigated extensively, its antimicrobial potential remains to be examined in detail. In the current study bactericidal assays were used to demonstrate that the secretome of dental pulp multipotent mesenchymal stromal cells (MSCs) has direct antibacterial activity against the archetypal Gram-positive and Gram-negative bacteria, *Staphylococcus aureus* and *Escherichia coli*, respectively, as well as the oral pathogens *Streptococcus mutans*, *Lactobacillus acidophilus*, and *Fusobacterium nucleatum*. Furthermore, a cytokine/growth factor array, enzyme-linked immunosorbent assays, and antibody blocking were used to show that cytokines and growth factors present in the dental pulp MSC secretome, including hepatocyte growth factor, angiopoietin-1, IL-6, and IL-8, contribute to this novel antibacterial activity. This study elucidated a novel and diverse antimicrobial secretome from human dental pulp MSCs, suggesting that these cells contribute to the antibacterial properties of the dental pulp. With this improved understanding of the secretome of dental pulp MSCs and its novel antibacterial activity, new evidence for the ability of the dental pulp to fight infection and restore functional competence is emerging, providing further support for the biological basis of pulpal repair and regeneration.

The pulpal response to caries is a complex process that ultimately aims to halt the carious lesion and protect the pulp from bacterial invasion.^1^ In slowly developing lesions, odontoblasts subjacent to the dentin may respond effectively to the caries ingress, to induce repair and reactionary dentine formation. However, in more rapidly developing carious lesions, the likely death of the odontoblasts means that new odontoblast-like cells, differentiated from stem/progenitor cells, are required to induce repair, regeneration, and tertiary dentine formation.

Given the importance of eliminating infection before repair and regeneration, constitutive expression of antibacterial factors in the dentin-pulp complex has been reported. Odontoblast-like cells express antimicrobial peptides, such as β-defensins,^2^^,^^3^ known to directly kill microorganisms and bind their bacterial toxins, such as lipopolysaccharide (reviewed by Mai et al^4^). Neutrophils recruited to the carious lesion site attempt to kill invading bacteria via neutrophil extracellular traps^5^ containing antimicrobial peptides, such as α-defensins, known to have broad specificity.^6^ The identification of additional antimicrobial factors that may contribute to the resolution of infection would provide a more complete picture of the restoration of tissue homeostasis and subsequent downstream repair/regenerative processes that are ultimately required to preserve the structure and function of the tooth.^7^

Although the regenerative capacity of dental pulp stem/progenitor cells has been studied extensively, their therapeutic potential in contributing to the resolution of pulpal infection remains unreported. Furthermore, although it is recognized that clearance of infection must precede pulpal regeneration, it has been assumed that human dental pulp stem cells contribute only to the pulp's regeneration, but not its antimicrobial potential. It has previously been reported that secreted products of human bone marrow–derived mesenchymal stromal cells (MSCs), including LL-37, contribute to their ability to inhibit bacterial growth.^8^ Indeed, progenitors from the oral mucosa lamina propria have also been shown to have antimicrobial properties.^9^ Adipose tissue–derived MSCs are also antimicrobial, and all human MSCs, regardless of their tissue origin, are potentially antimicrobial and may have functional consequences in infection.^10^ Although dental pulp fibroblasts are capable of inhibiting bacterial growth,^11^ to date there has been no indication that the dental pulp MSC secretome is antimicrobial. We hypothesized that dental pulp MSCs secrete factors capable of inhibiting bacterial growth, thus enabling them to contribute to the overall defense strategy of the dental pulp.

## Materials and Methods

### Cell Culture

Human dental pulp cells (from three biological donors) were harvested from immature permanent third molar teeth,^12^ in accordance with French ethics legislation and covered by the Office for Research Ethics Committees (Northern Ireland). Dental pulp cells were cultured in minimal essential media-α, supplemented with 10% fetal bovine serum and 2 mmol/L L-glutamine, in a 37°C incubator with 5% CO_2_. Stro-1–positive dental pulp multipotent MSCs (subsequently referred to as dental pulp MSCs) were isolated from dental pulp cell cultures at passage 2 or 3, as previously described,^13^ by magnetically activated cell sorting using mouse anti–Stro-1 IgM conjugated to anti-mouse IgM magnetic Dynabeads (Life Technologies/Thermo Fisher Scientific, Dublin, Ireland), according to the manufacturer's instructions.

### Cell Characterization by Flow Cytometry and Differentiation Potential

Dental pulp MSCs were stained for expression of CD90, CD73, CD105, CD44, CD45, CD34, CD11b, CD19, and human leukocyte antigen-DR using the BD Stemflow Human MSC Analysis Kit (BD Biosciences, Wokingham, UK), according to the manufacturer's instructions. Flow cytometry was performed on the Attune NxT Acoustic Focusing Cytometer (Thermo Fisher Scientific, Carlsbad, CA), with data analysis using FlowJo 10.4 software (BD Biosciences).

The trilineage (adipogenic, chondrogenic, and osteogenic) differentiation potential of the cells was confirmed following exposure to adipogenic, chondrogenic, and osteogenic media for 2 to 3 weeks and subsequent staining with Oil Red O, Safranin O, and Alizarin Red, respectively, to identify the cell phenotypes.^14^

### Bacterial Culture

*Escherichia coli* and *Staphylococcus aureus* were selected for this study based on their use in previous work describing the antimicrobial efficacy of stem cells from bone marrow, adipose tissue, and umbilical cord blood.^8^^,^^10^^,^^15^^,^^16^ To demonstrate that the dental pulp MSC secretome provides defense against relevant oral pathogens, *Streptococcus mutans*, *Lactobacillus acidophilus*, and *Fusobacterium nucleatum* were also studied. All microorganisms were cultured at 37°C.

Briefly, *E. coli* ATCC (Manassas, VA) 25922, *S. aureus* ATCC 25923, and *S. mutans* National Collection of Type Cultures (NCTC) 10449 (Salisbury, UK) were cultured aerobically on Columbia blood agar plates (Southern Group Laboratories, Corby, UK), whereas *L. acidophilus* ATCC 4356 was cultured under microaerophilic conditions (candle jar) on deMan, Rogosa, and Sharpe agar (Oxoid, Basingstoke, UK) and *F. nucleatum* NCTC 10562 was cultured anaerobically on fastidious anaerobe agar (Oxoid). Overnight cultures of *E. coli*, *S. aureus*, and *S. mutans* were prepared in Mueller Hinton broth, *L. acidophilus* was grown in deMan, Rogosa, and Sharpe broth (Oxoid), and *F. nucleatum* was grown anaerobically in fastidious anaerobe broth (Oxoid). Following overnight culture, 10 mL of fresh broth was inoculated with 100 μL of the overnight culture and grown to mid–logarithmic phase with gentle shaking. The approximate number of colony-forming units per mL in the mid-logarithmic cultures was obtained by measuring the OD at 600 nm and comparing with established growth curves. Having reached mid-logarithmic phase, cultures were washed and resuspended in sterile sodium phosphate buffer, pH 7.4, before use in experiments.

### Antibacterial Assays

Dental pulp MSCs were seeded in 6-well plates (3 × 10^5^ cells/mL) and grown to confluency. Cells were incubated for 24 hours in serum-free minimal essential media-α (supplemented with 2 mmol/L L-glutamine), and the conditioned medium containing the dental pulp MSC secretome was collected and frozen at −20°C until required for testing antibacterial efficacy.

A modified microbroth dilution susceptibility assay^8^^,^^17^ was used to assess the antibacterial effects of the dental pulp MSC secretome. Individual cytokines/growth factors found to be abundantly produced by dental pulp MSCs were also tested for their antibacterial activity using this assay. Briefly, 50-μL aliquots^18^ of the appropriate diluent (minimal essential media-α or sodium phosphate buffer, pH 7.4) or test substance (dental pulp MSC conditioned media or cytokines/growth factors in sodium phosphate buffer, pH 7.4) were added to 96-well plates (Nunc; Thermo Fisher Scientific, Dublin, Ireland), inoculated with *S. aureus*, *E. coli*, *S. mutans*, *L. acidophilus*, or *F. nucleatum* (50 μL; 2 × 10^3^ colony-forming units/mL)^8^ in sodium phosphate buffer, pH 7.4, and incubated at 37°C for 2 hours. Culture medium from each well was serially diluted with sterile phosphate-buffered saline and plated on appropriate agar plates (as outlined above) for enumeration^19^ following overnight incubation at 37°C.

### Cytokine/Growth Factor Multiplex Array

A multiplex array for the simultaneous detection of 43 cytokines and growth factors (Abcam, Cambridge, UK) was employed to analyze the dental pulp MSC secretome. Conditioned media (containing the dental pulp MSC secretome) were collected from the cells following 24 hours of serum starvation. The array was performed according to the manufacturer's protocol, and membranes were imaged using a G:BOX (Syngene, Cambridge, UK). Images were semiquantified using the ImageJ software version 1.5 (NIH, Bethesda, MD), image analysis program, with a microarray plugin (ImageJ bundled with 64-bit Java 1.8.0_172; downloaded from *https://imagej.nih.gov/ij*, last accessed September 1, 2021). The integrated density of each spot was measured, and the final integrated density was calculated as described by the manufacturer to allow comparison of the integrated densities of array spots over multiple membranes.

### Enzyme-Linked Immunosorbent Assay

As the multiplex array did not contain an antibody for detection of hepatocyte growth factor (HGF), it was measured in the dental pulp MSC conditioned media using a DuoSet ELISA DY294 (R&D Systems, Minneapolis, MN). Given their antimicrobial efficacy (reported below), the concentrations of IL-6, IL-8, and angiopoietin-1 (ANGPT-1) were also measured in the dental pulp MSC secretome using DuoSet ELISAs DY206, DY208, and DY923, respectively (R&D Systems).

### RNA Extraction, cDNA Synthesis, and Real-Time Quantitative PCR

Dental pulp MSCs were grown to confluency in 6-well plates, as described for the antibacterial assay, using serum-free conditions for 24 hours before RNA harvesting. RNA was extracted using the RNeasy Plus Mini kit (Qiagen, Hilden, Germany), according to the manufacturer's instructions. RNA concentration and purity were measured by NanoDrop (Thermo Fisher Scientific, Dublin, Ireland). cDNA was synthesized from 100 ng of extracted RNA using the SuperScript VILO cDNA synthesis kit (Thermo Fisher Scientific, Dublin, Ireland), according to the manufacturer's instructions. Real-time quantitative PCR, to determine the expression levels of the antimicrobial peptides cathelicidin antimicrobial peptide (*CAMP*), defensin β 103 (*DEFB103*), defensin β 4A (*DEFB4A*), and lipocalin-2 (*LCN2*) and the reference genes [glucuronidase β (*GusB*) and β-2-microglobulin (*B2M*)], was performed using the TaqMan Universal Mastermix II with UNG (Applied Biosystems, Waltham, MA) protocol, with a reaction mixture of 20 μL. Details of the primers (TaqMan Gene Expression Assays; Applied Biosystems) used are given in Table 1, and the real-time quantitative PCR conditions are detailed in Table 2.

**Table 1:** TaqMan Gene Expression Assay Primer Details

Gene	Reference no.	Chromosome no.	Probe sits on exon boundary	Base position	Amplicon length, bp
*CAMP*	Hs00189038_m1	3	1–2	365	86
*DEFB103*	Hs00218678_m1	8	1–2	276	93
*DEFB4A*	Hs00823638_m1	8	1–2	204	86
*LCN2*	Hs01008571_m1	9	5–6	665	61
*GusB*	Hs00939627_m1	7	8–9	1522	96
*B2M*	Hs00984230_m1	15	3–4	431	81

**Table 2:** Thermal Profile for qPCR Conditions

Segment	Thermal profile	Cycles, *N*
1	2 Minutes at 50°C	1
2	10 Minutes at 95°C	1
3	30 Seconds at 95°C and 1 minute at 60°C	40

qPCR, real-time quantitative PCR.

### Sytox Green Membrane Permeabilization Assay

To determine whether antibacterial activity was achieved via membrane permeabilization, a Sytox Green assay (Thermo Fisher Scientific, Dublin Ireland) was performed, as previously described.^20^ Briefly, cultures of mid–logarithmic phase *S. aureus* and *E. coli* were adjusted to an OD of 0.7 at 600 nm, using 5% Mueller-Hinton broth. A 50-μL aliquot of the bacterial suspension was added to each well of a 96-well black flat bottom plate (Corning Costar; Sigma-Aldrich, Poole, UK), and the test cytokine/growth factor (10 ng/mL; diluted in 5% Mueller-Hinton broth) was added. Sytox Green (ThermoFisher; to 5 μmol/L) was then added to each well. The plate was incubated at 37°C for 2 hours, protected from light. Untreated bacteria served as negative controls, and bacteria permeabilized with 70% isopropyl alcohol served as positive controls. Additional wells containing 5% Mueller-Hinton broth and Sytox Green were included to quantify potential background fluorescence. The plate was read on a Genios microplate reader (Tecan, Männedorf, Switzerland) with excitation at 480 nm and an emission at 535 nm.

### Blocking Experiments with Anti-HGF Antibody

Proof-of-principle experiments were undertaken to determine the effect of blocking the effect of HGF on the overall antibacterial activity of the dental pulp MSC secretome against Gram-positive and Gram-negative bacteria. Briefly, the conditioned medium containing the dental pulp MSC secretome was treated with anti-HGF blocking antibody (Bio-Techne, Abingdon, UK) for 2 hours (1 μg/mL) before testing for antibacterial activity against *S. aureus*, *E. coli*, *L. acidophilus*, or *F. nucleatum* (as outlined above).

### Statistical Analysis

Statistical comparisons were performed using Prism version 8.4.2 (GraphPad Software, San Diego, CA). Data were analyzed from a minimum of three independent experiments (with at least three replicates per experiment). Data that passed the Shapiro-Wilk test for normality distribution were analyzed by the *t*-test or one-way analysis of variance with Dunnett or Holm-Sidak post hoc testing; otherwise, data were analyzed nonparametrically by *U*-test or Kruskal-Wallis one-way analysis of variance with the Dunn correction for multiple comparison testing, as indicated in the figure legends. In all experiments, *P* ≤ 0.05 was considered statistically significant.

## Results

### Flow Cytometry Analysis of Stro-1 Sorted Dental Pulp MSCs

Dental pulp MSCs were positive for CD90, CD73, CD105, and CD44 (Supplemental Figure S1, A–D), and negative for CD34, CD11b, CD19, and human leukocyte antigen-DR (Supplemental Figure S1E). Cells were also shown to have the ability to differentiate to adipogenic, chondrogenic, and osteogenic lineages *in vitro* (Supplemental Figure S2).

### The Human Dental Pulp MSC Secretome Is Antibacterial

**Figure 1:**
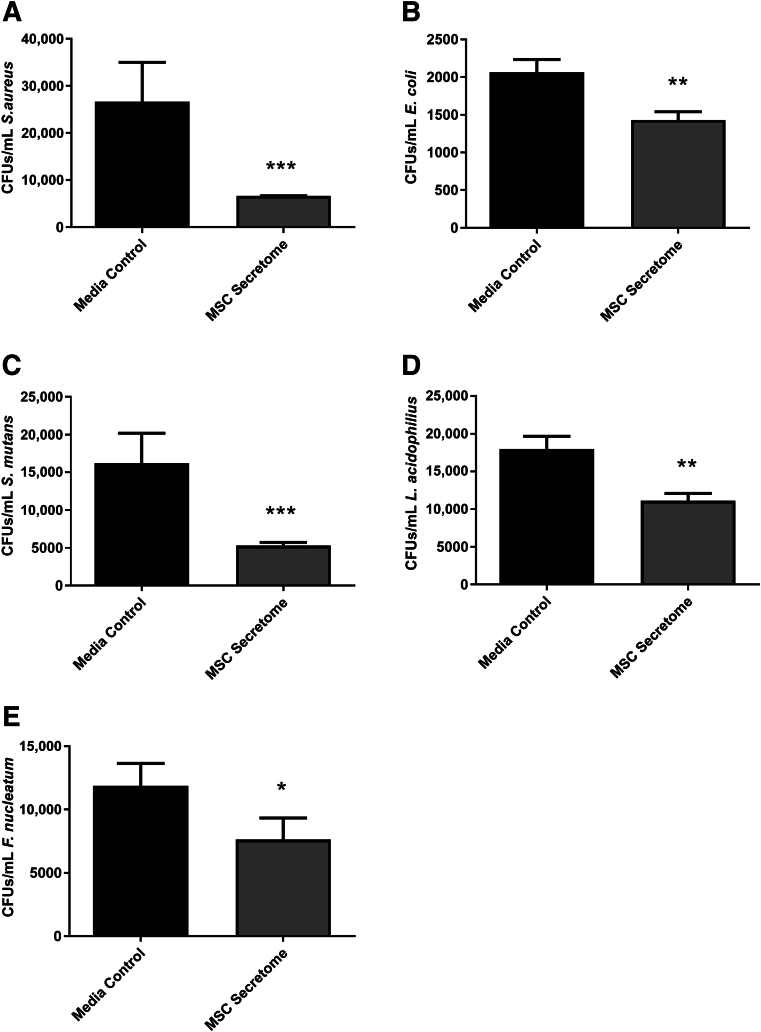
Efficacy of conditioned media (secretome) from dental pulp mesenchymal stromal cells (MSCs) against *Staphylococcus aureus* (**A**), *Escherichia coli* (**B**), *Streptococcus mutans* (**C**), *Lactobacillus acidophilus* (**D**), and *Fusobacterium nucleatum* (**E**). Conditioned media showed significant antibacterial activity against *S. aureus*, *E. coli*, *S. mutans*, *L. acidophilus*, and *F. nucleatum* when compared with media control. Results from three independent experiments. *Staphylococcus aureus*, *E. coli*, and *L. acidophilus* data analyzed by *t*-test; *S. mutans* and *F. nucleatum* data analyzed by *U*-test. ∗*P* < 0.05, ∗∗*P* < 0.01, and ∗∗∗*P* < 0.001. CFU, colony-forming unit.

To determine the antibacterial activity of the human dental pulp MSC secretome, conditioned medium was collected from dental pulp MSCs and used to treat broth cultures of *S. aureus*, *E. coli*, *S. mutans*, *L. acidophilus*, and *F. nucleatum*. The number of colony-forming units of *S. aureus*, *E. coli*, *S. mutans*, *L. acidophilus*, and *F. nucleatum* were all shown to be significantly reduced following treatment with dental pulp MSC conditioned media (Figure 1).

### Gene Expression of Classic Antimicrobial Peptides Is Low in Dental Pulp MSCs

Given that the dental pulp MSC secretome displayed antibacterial activity against both Gram-positive and Gram-negative bacteria, it was hypothesized that dental pulp MSCs expressed one or more antimicrobial peptides (AMPs). To test this hypothesis, the mRNA expression levels of four major AMPs (LL-37, β-defensin 2, β-defensin 3, and lipocalin) were analyzed by real-time quantitative PCR. For LL-37, β-defensin 2, and β-defensin 3, no detectable mRNA levels of any of the peptides were found. Lipocalin-2 was lowly expressed, with cycle threshold values consistently around 38. Average cycle threshold values for each of the AMPs and the housekeeping genes can be found in Supplemental Table S1.

### Growth Factor/Cytokine Analysis of the Dental Pulp MSC Secretome

To further investigate soluble factors that could be responsible for the observed antimicrobial activity, dental pulp MSC conditioned media were analyzed by an antibody-based multiplex cytokine array. Of the 43 growth factors/cytokines analyzed by the Abcam array, 19 had an integrated density >1 in the dental pulp MSC secretome, as determined by ImageJ software (Table 3). These were angiogenin, growth-regulated α protein, interferon-γ, IL-6, IL-8, leptin, monocyte chemoattractant protein-1, placental growth factor, regulated upon activation normal T cell expressed and secreted, transforming growth factor-β, tissue inhibitor of matrix metalloproteinases-1 (TIMP-1), TIMP-2, thrombopoietin, vascular endothelial growth factor, ANGPT-1, ANGPT-2, plasminogen, matrix metalloproteinase-1, and urokinase plasminogen activator surface receptor. Of the 19 detectable growth factors/cytokines on the array, 8 were found to be the most highly expressed, with an integrated density >10 (growth-regulated α protein, IL-6, IL-8, monocyte chemoattractant protein-1, TIMP-1, TIMP-2, ANGPT-1, and ANGPT-2) (Table 3).

**Table 3:** A Total of 43 Growth Factors/Cytokines Were Analyzed by Multiplex Array

Array abbreviation	Full name	Array integrated density
GRO-α	Growth-related α protein	57.38
MCP-1	Monocyte chemoattractant protein-1	54.90
TIMP-2	Tissue inhibitor of matrix metalloproteinase-2	32.58
IL-6	Interleukin-6	31.00
TIMP-1	Tissue inhibitor of matrix metalloproteinase-1	26.26
ANGPT1	Angiopoietin-1	18.78
IL-8	Interleukin-8	13.97
ANGPT2	Angiopoietin-2	10.63
TGF-β	Transforming growth factor-β	6.76
ANG	Angiogenin	4.63
MMP-1	Matrix metalloproteinase-1	4.44
VEGF	Vascular endothelial growth factor	4.38
RANTES	Regulated upon activation normal T cell expressed and secreted	3.53
THPO	Thrombopoietin	1.83
Leptin	Leptin	1.55
PLG	Plasminogen	1.19
uPAR	Urokinase-type plasminogen activator receptor	1.18
IFN-γ	Interferon-γ	1.06
PLGF	Placental growth factor	1.03

Of these, 19 had integrated densities >1 in the secretome of the dental pulp mesenchymal stromal cells and are tabulated above, in order of expression level. The first eight growth factors/cytokines tabulated had integrated densities >10.

**Figure 2:**
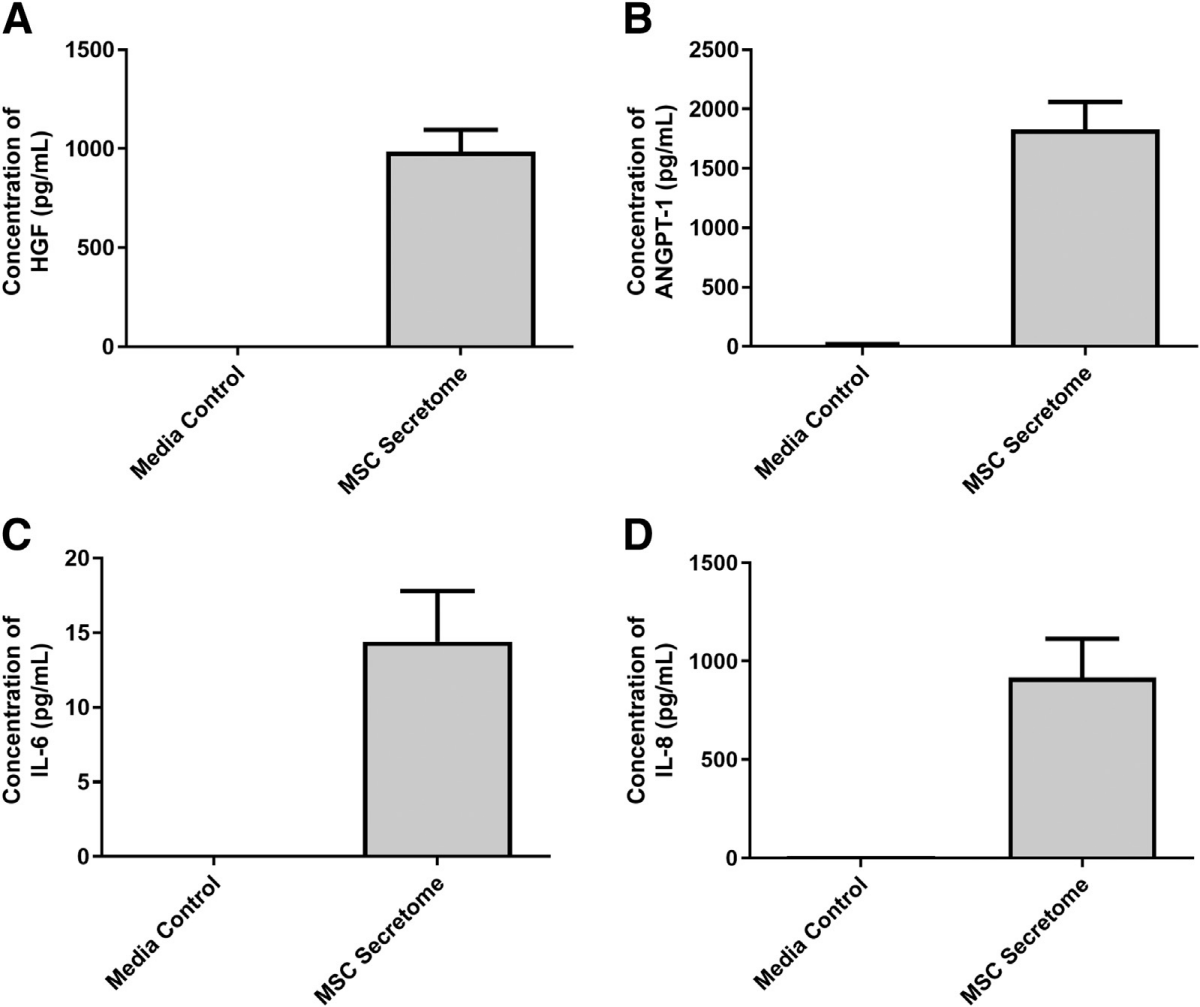
The concentration of hepatocyte growth factor (HGF; **A**), angiopoietin-1 (ANGPT-1; **B**), IL-6 (**C**), and IL-8 (**D**) in the dental pulp mesenchymal stromal cell (MSC) secretome, measured by enzyme-linked immunosorbent assay.

The array did not contain an antibody for the detection of HGF, which is known to be highly expressed in the dental pulp^21^^,^^22^ and by MSCs.^23^^,^^24^ The levels of HGF in the dental pulp MSC secretome were measured by enzyme-linked immunosorbent assay. Dental pulp MSCs expressed HGF in the range of 800 to 1200 pg/mL (mean, 984 pg/mL) (Figure 2A). Given their antimicrobial efficacy, the levels of ANGPT-1, IL-6, and IL-8 were also measured by enzyme-linked immunosorbent assay; and their respective concentration ranges were found to be as follows: ANGPT-1, 1500 to 2300 pg/mL (mean, 1827 pg/mL) (Figure 2B); IL-6, 10 to 22 pg/mL (mean, 14 pg/mL) (Figure 2C); and IL-8, 330 to 1500 pg/mL (mean, 916 pg/mL) (Figure 2D), confirming their expression, as observed in the growth factor/cytokine array.

### Antibacterial Activity of Growth Factors/Cytokines

**Figure 3:**
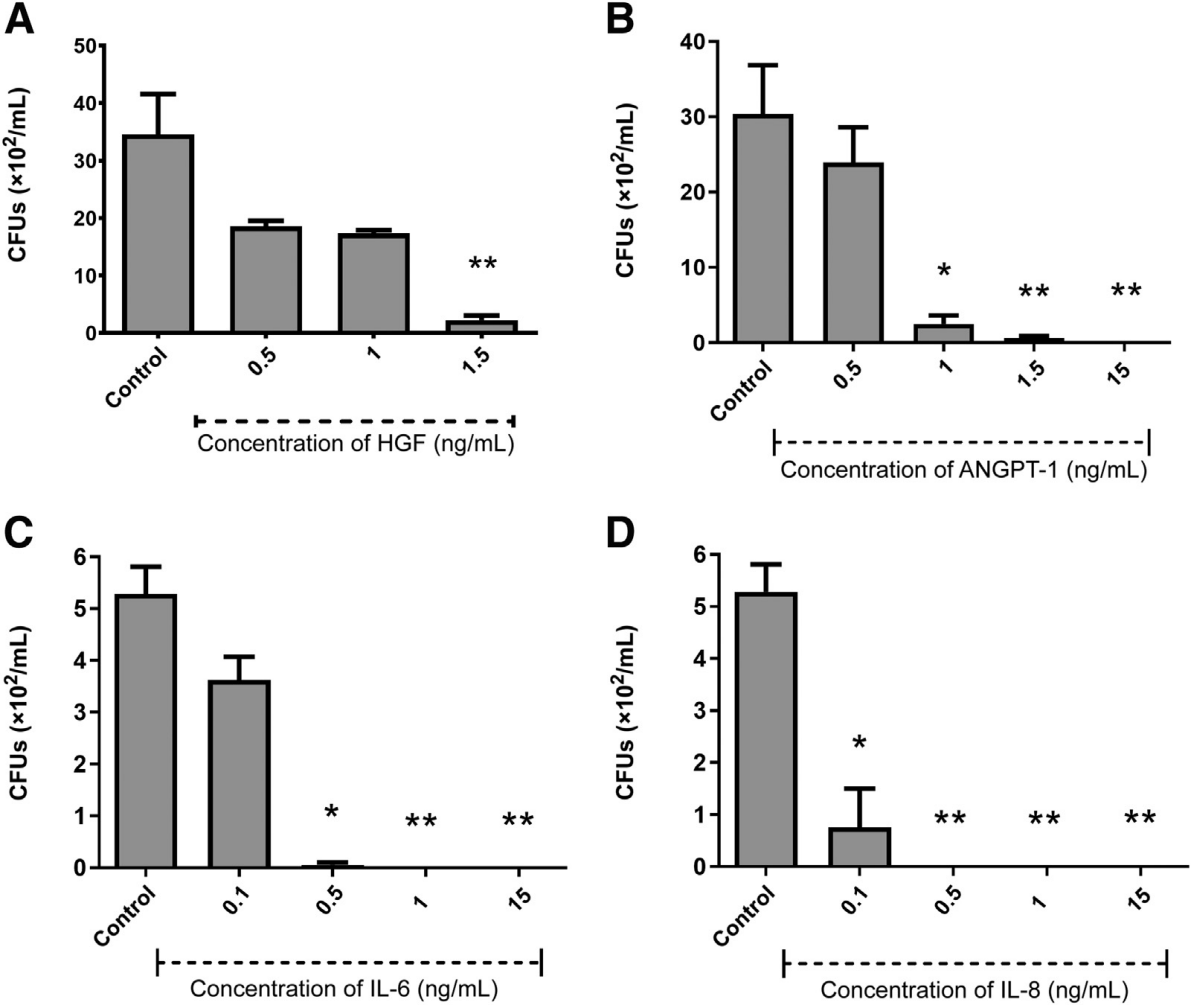
Antimicrobial efficacy of growth factors/cytokines against *Escherichia coli* hepatocyte growth factor (HGF; **A**), angiopoietin-1 (ANGPT-1; **B**), IL-6 (**C**), and IL-8 (**D**). Results from three independent experiments are presented. Data were analyzed by Kruskal-Wallis one-way analysis of variance with Dunn correction for multiple comparison testing. ∗*P* < 0.05, ∗∗*P* < 0.01. CFU, colony-forming unit.

**Figure 4:**
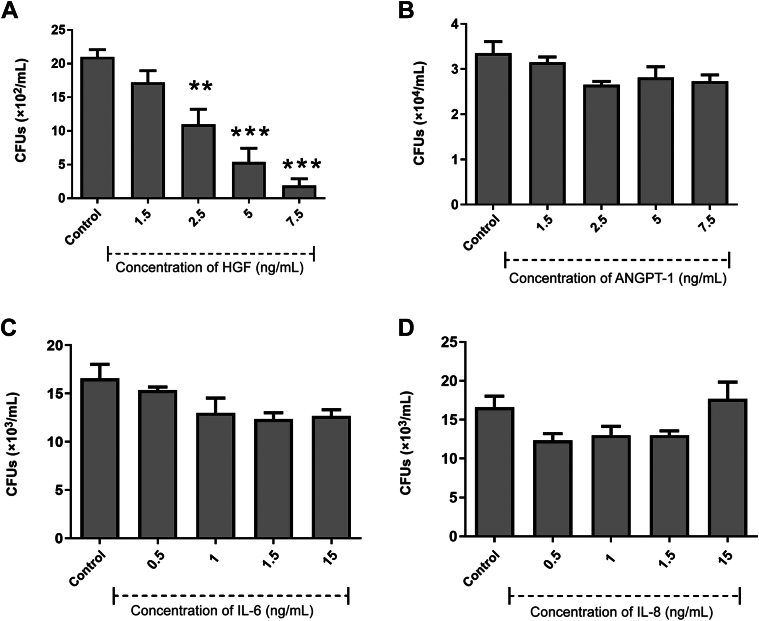
Antimicrobial efficacy of growth factors/cytokines against *Staphylococcus aureus* hepatocyte growth factor (HGF; **A**), angiopoietin-1 (ANGPT-1; **B**), IL-6 (**C**), and IL-8 (**D**). Results from three independent experiments are presented. Data were analyzed by analysis of variance with Dunnett correction for multiple comparison testing. ∗∗*P* < 0.01, ∗∗∗*P* < 0.001. CFU, colony-forming unit.

**Figure 5:**
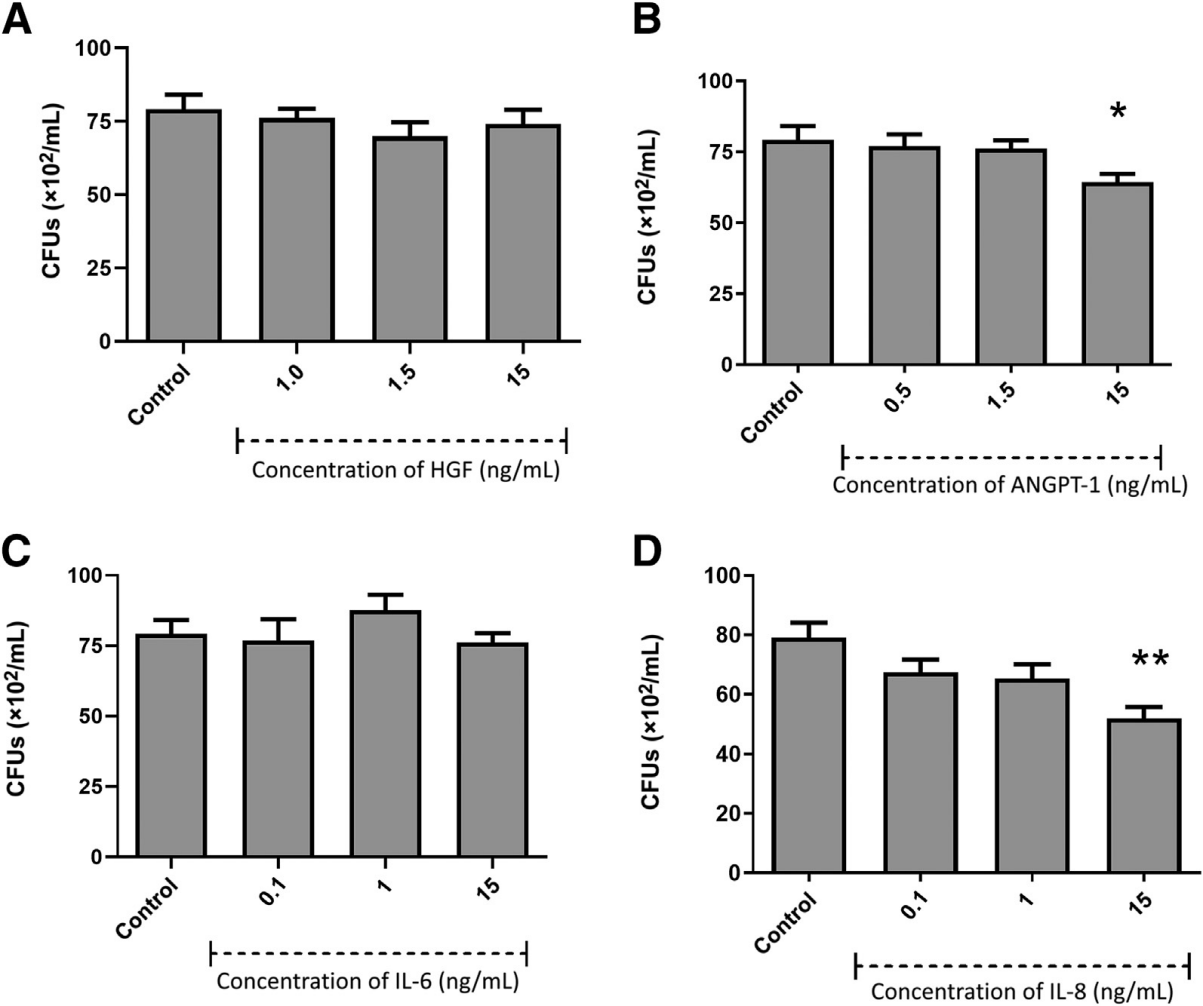
Antimicrobial efficacy of growth factors/cytokines against *Streptococcus mutans* hepatocyte growth factor (HGF; **A**), angiopoietin-1 (ANGPT-1; **B**), IL-6 (**C**), and IL-8 (**D**). Results from three independent experiments are presented. Data were analyzed by Kruskal-Wallis one-way analysis of variance with Dunn correction for multiple comparison testing. ∗*P* < 0.05, ∗∗*P* < 0.01. CFU, colony-forming unit.

**Figure 6:**
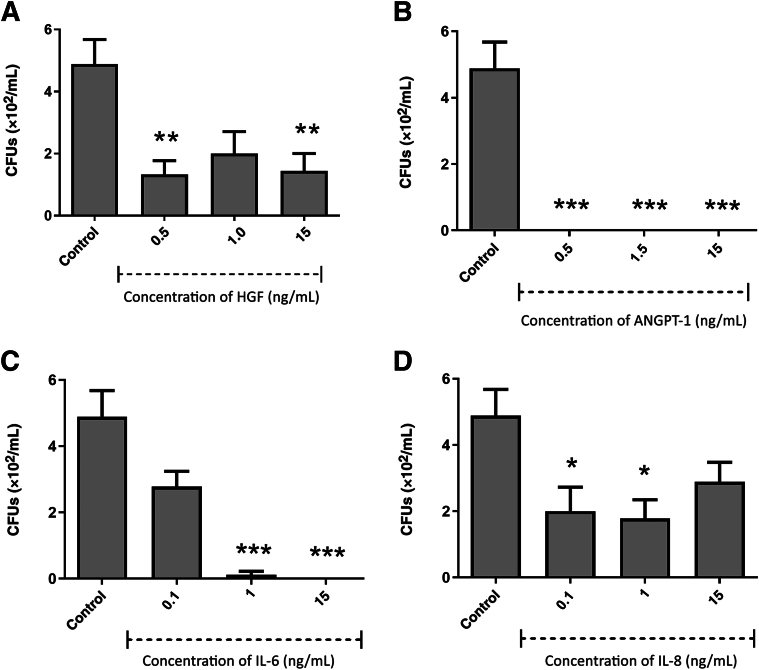
Antimicrobial efficacy of growth factors/cytokines against *Lactobacillus acidophilus* hepatocyte growth factor (HGF; **A**), angiopoietin-1 (ANGPT-1; **B**), IL-6 (**C**), and IL-8 (**D**). Results from three independent experiments are presented. Data were analyzed by Kruskal-Wallis one-way analysis of variance with Dunn correction for multiple comparison testing. ∗*P* < 0.05, ∗∗*P* < 0.01, and ∗∗∗*P* < 0.001. CFU, colony-forming unit.

**Figure 7:**
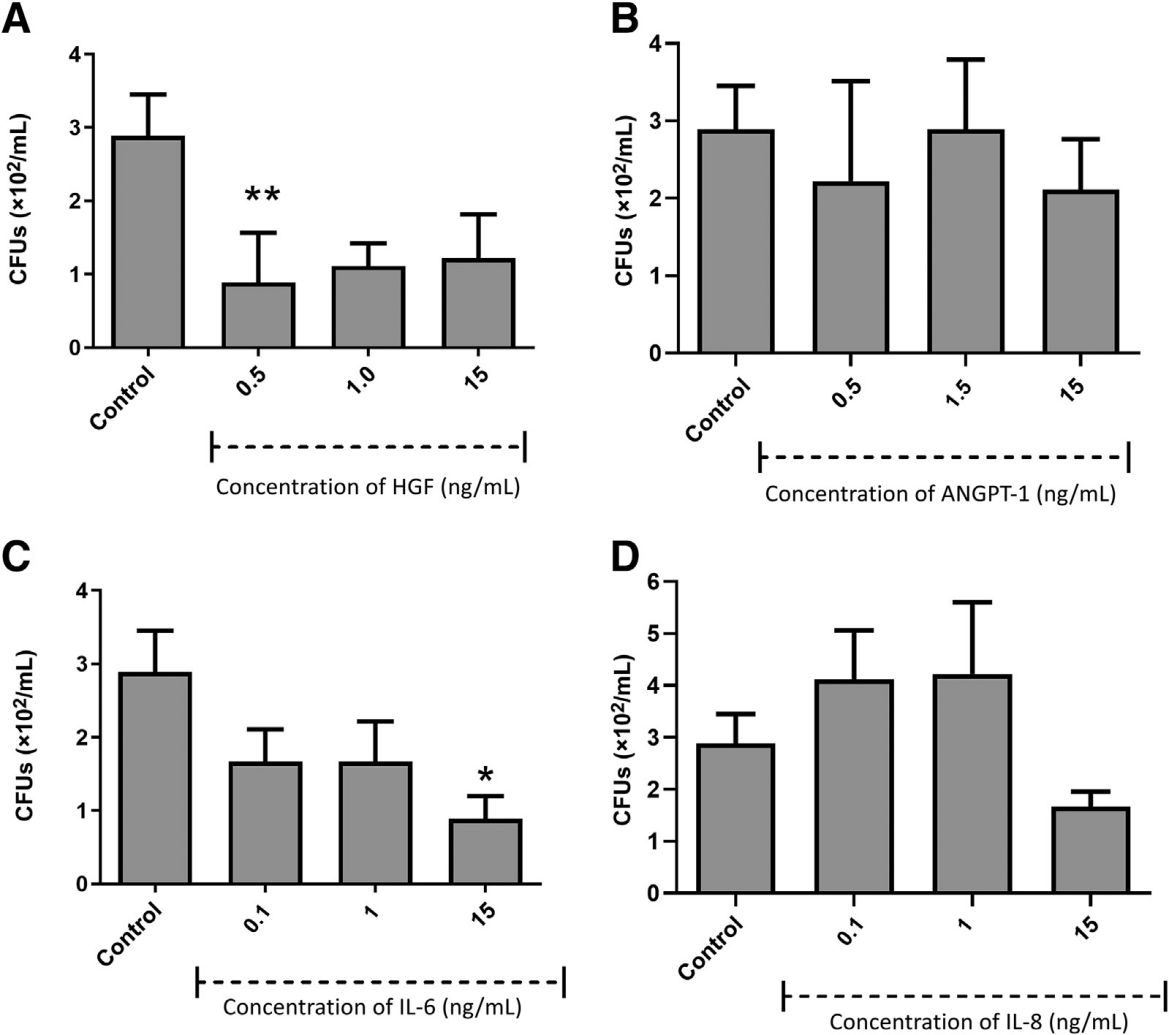
Antimicrobial efficacy of growth factors/cytokines against *Fusobacterium nucleatum* hepatocyte growth factor (HGF; **A**), angiopoietin-1 (ANGPT-1; **B**), IL-6 (**C**), and IL-8 (**D**). Results from three independent experiments are presented. Data were analyzed by Kruskal-Wallis one-way analysis of variance with Dunn correction for multiple comparison testing. ∗*P* < 0.05, ∗∗*P* < 0.01. CFU, colony-forming unit.

Given the low gene expression levels of classic antimicrobial peptides in dental pulp MSCs, it was hypothesized that one or more of the growth factors/cytokines produced in abundance by the dental pulp MSCs may be responsible for the antibacterial properties of the secretome. We showed that four of the growth factors/cytokines (HGF, ANGPT-1, IL-6, and IL-8) had variable antibacterial activity against *E. coli* (Figure 3), *S. aureus* (Figure 4), *S. mutans* (Figure 5), *L. acidophilus* (Figure 6), and *F. nucleatum* (Figure 7). However, monocyte chemoattractant protein-1, growth-regulated α protein, TIMP-1, and TIMP-2 did not show antibacterial activity against any of the bacteria studied at the concentrations tested (0.5 to 15 ng/mL) (results not shown).

HGF displayed significant bactericidal activity against the archetypal Gram-negative bacterium *E. coli* (Figure 3) at 1.5 ng/mL. ANGPT-1 displayed significant bactericidal activity at a range of concentrations from 1 to 15 ng/mL. IL-6 was shown to have significant antibacterial activity at concentrations from 0.5 to 15 ng/mL. IL-8 showed significant antibacterial activity against *E. coli* at all concentrations in the range from 0.1 to 15 ng/mL. When the same growth factors/cytokines were also tested for potential antibacterial activity against the archetypal Gram-positive bacterium, *S. aureus* (Figure 4) HGF was the only growth factor that showed significant bactericidal action against *S. aureus* (at concentrations from 2.5 to 7.5 ng/mL).

The growth factors/cytokines also had variable antibacterial activity against the oral pathogens *S. mutans*, *L. acidophilus*, and *F. nucleatum*. ANGPT-1 and IL-8 displayed significant bactericidal activity against *S. mutans* at a concentration of 15 ng/mL (Figure 5). *Lactobacillus acidophilus* (Figure 6) was susceptible to HGF at 0.5 and 15 ng/mL. ANGPT-1 showed significant bactericidal activity to *L. acidophilus* at all concentrations tested (0.5 to 15 ng/mL). IL-6 was shown to have significant antibacterial activity against *L. acidophilus* at 1 and 15 ng/mL, whereas IL-8 was significantly antibacterial at 0.1 and 1 ng/mL. HGF was shown to be efficacious against *F. nucleatum* at a concentration of 0.5 ng/mL, and IL-6 was antibacterial only at a concentration of 15 ng/mL (Figure 7).

### HGF Contributes to the Antibacterial Activity of the Dental Pulp MSC Secretome

**Figure 8:**
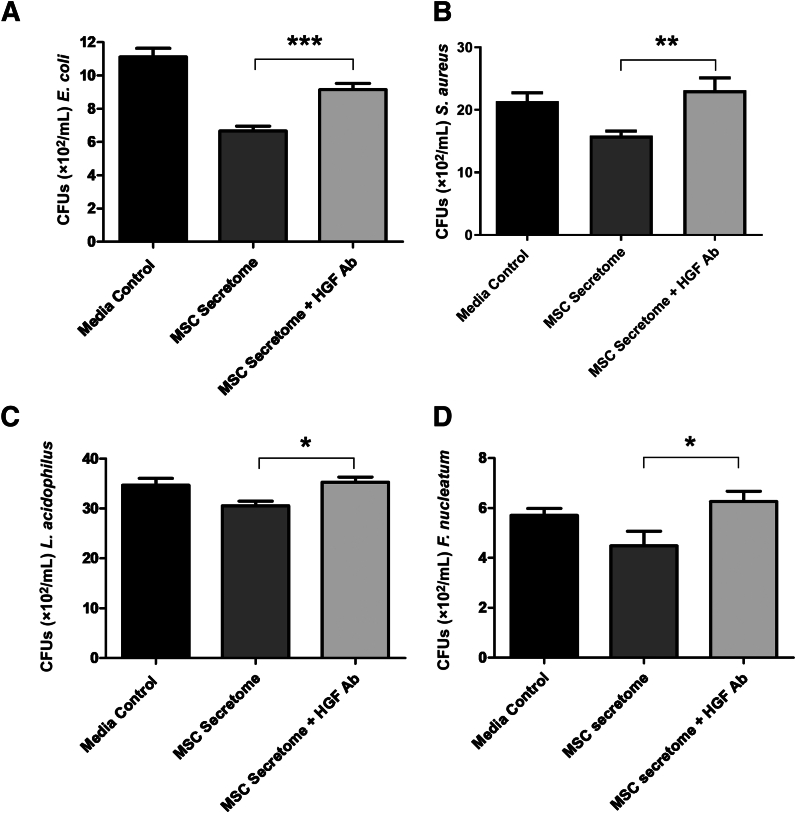
Blocking with anti–hepatocyte growth factor (HGF) antibody (Ab) reduced the antibacterial efficacy of the dental pulp mesenchymal stromal cell (MSC) secretome (conditioned media) against *Escherichia coli* (**A**), *Staphylococcus aureus* (**B**), *Lactobacillus acidophilus* (**C**), and *Fusobacterium nucleatum* (**D**). Results from three independent experiments are presented. Data for *E. coli* were analyzed by Kruskal-Wallis one-way analysis of variance with Dunn correction for multiple comparison testing. Data for *S. aureus*, *L. acidophilus*, and *F. nucleatum* were analyzed by one-way analysis of variance with Holm-Sidak correction for multiple comparison testing. All comparisons were made between the MSC secretome and the MSC secretome + HGF blocking antibody. ∗*P* < 0.05, ∗∗*P* < 0.01, and ∗∗∗*P* < 0.001. CFU, colony-forming unit.

Because HGF was one of the growth factors to display antimicrobial activity against both Gram-positive and Gram-negative bacteria (*E. coli*, *S. aureus*, *L. acidophilus*, and *F. nucleatum*), further confirmation of its contribution to the antibacterial activity of the dental pulp MSC secretome was provided by blocking experiments in the presence of anti-HGF antibodies. Results showed that anti-HGF antibodies significantly reduced the antibacterial activity of the conditioned media, suggesting that HGF was directly responsible for at least a component of the dental pulp MSC secretome's antibacterial activity in these bacteria (Figure 8). Blocking experiments were not undertaken for *S. mutans*, as HGF did not show significant antibacterial activity against this microorganism (Figure 5).

### Membrane Permeabilization of *E. coli* and *S. aureus* as a Potential Mechanism of Antibacterial Activity

**Figure 9:**
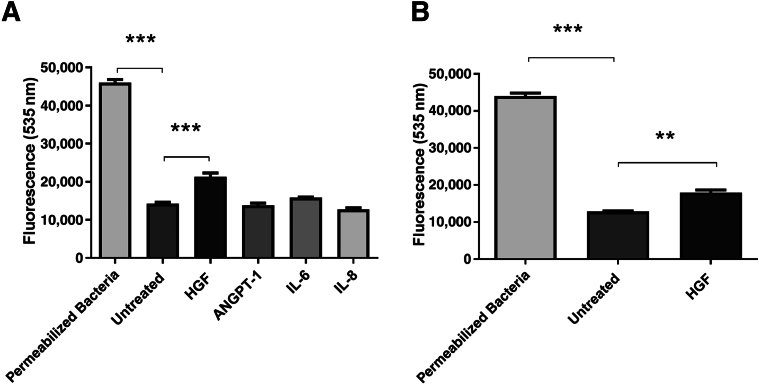
Sytox Green permeabilization assay with *Escherichia coli* (**A**) or *Staphylococcus aureus* (**B**). Relative fluorescence is reported at 535 nm after treatment with hepatocyte growth factor (HGF), angiopoietin-1 (ANGPT-1), IL-6, or IL-8. Significantly increased fluorescence was observed following HGF treatment of both *E. coli* and *S. aureus* compared with untreated bacteria. Treatment of bacteria with 70% isopropyl alcohol served to permeabilize bacterial membranes and act as positive controls. Results from three independent experiments. Kruskal-Wallis one-way analysis of variance with Dunn correction for multiple comparison testing was performed. ∗∗*P* < 0.01, ∗∗∗*P* < 0.001.

Most classic AMPs exert their bactericidal effects through bacterial membrane disruption. Whether the cytokines found to be antibacterial in this work may have similar mechanisms of action is not known. Of the growth factors/cytokines shown to be antibacterial against *E. coli* (HGF, ANGPT-1, IL-6, and IL-8), only HGF displayed significant membrane permeabilization effects (Figure 9A). Because HGF was the only cytokine found to possess antibacterial activity against *S. aureus*, only its membrane permeabilization activity was tested. HGF was also shown to have significant membrane permeabilization effects on *S. aureus* (Figure 9B).

## Discussion

Despite our understanding of the regeneration capacity of dental pulp stem/progenitor cells, the antimicrobial potential of their secretome has not previously been investigated. This study used Stro-1^+^ dental pulp MSCs, verified for expression of stem cell markers (CD73, CD90, and CD105, and negative for CD45, CD34, CD11b, CD19, and human leukocyte antigen-DR), and showed, for the first time, that the Stro-1^+^ dental pulp MSC secretome possesses inherent antibacterial activity. Progenitor/stem cells derived from several anatomic sources are antimicrobial.^8^, ^9^, ^10^^,^^15^^,^^16^ However, the factors responsible for the antibacterial activity are variable, depending on the cell source. Bone marrow MSCs have antibacterial activity against both *E. coli*^8^ and *S. aureus*,^8^^,^^10^ mediated principally by LL-37. The antibacterial activity of human umbilical cord blood MSCs against *E. coli* is mediated by β-defensin-2.^16^ In addition to classic AMPs, several MSC types secrete additional factors with antimicrobial activity. Bone marrow MSCs secrete indoleamine 2,3-dioxygenase, with antibacterial activity against *S. aureus*,^25^ and adipose stem cells secrete regenerating islet-derived III γ, also with antibacterial activity against *S. aureus*.^15^ Furthermore, oral mucosal lamina propria progenitors exert antibacterial activity against a range of pathogens via the secretion of osteoprotegerin and haptoglobin.^9^ To our knowledge, the antimicrobial activity of MSC/progenitor stem cell secretome against *S. mutans*, *L. acidophilus*, or *F. nucleatum* has not been reported previously.

Many AMPs have immunomodulatory functions, resembling those conventionally described for growth factors and cytokines.^26^ Similarly, soluble factors, such as indoleamine 2,3-dioxygenase, regenerating islet-derived III γ haptoglobin, and osteoprotegerin (which acts like a cytokine), have been reported to have direct bactericidal effects.^9^^,^^15^^,^^25^ The current study shows that the cytokines IL-6 and IL-8, along with the growth factors HGF and ANGPT-1, all four of which are present in the dental pulp MSC secretome, possess variable antibacterial activity against *E. coli*, *S. aureus*, *S. mutans*, *L. acidophilus,* and *F. nucleatum*. It is important to recognize that the levels of growth factors/cytokines measured in the dental pulp MSC secretome are higher than those shown to display significant antibacterial activity *in vitro*, thereby supporting their potential physiological role in contributing to defense of the dental pulp. Furthermore, to demonstrate antibacterial functionality of one of these factors (HGF) in the MSC secretome, blocking experiments in the presence of anti-HGF antibodies were undertaken. The results showed that blocking antibodies significantly reduced the antibacterial efficacy of HGF, supporting a role for HGF in contributing to the antibacterial activity of dental pulp MSC secretome and providing proof-of-principle support for the direct antibacterial efficacy of the growth factors/cytokines.

The amino acid sequences of IL-6, IL-8, HGF, and ANGPT-1 were predicted to have bactericidal regions using the online tool AMPA (*http://tcoffee.crg.cat/apps/ampa*, last accessed September 2, 2021), an antimicrobial sequence scanner for predicting active regions in antimicrobial proteins.^27^ Although the current data suggest that HGF has membrane disrupting potential that may contribute to its mode of antibacterial action, ANGPT-1, IL-6, and IL-8 do not appear to exert antibacterial effects through this mechanism of action. It is, however, possible that ANGPT-1, IL-6, and IL-8 may act on specific intracellular or extracellular targets. Several antibacterial agents, such as the proline-arginine–rich peptide, PR-39, indolicidin, α-defensin-1, and buforin II, have been shown to inhibit bacterial growth by disrupting the synthesis of DNA, RNA, and/or proteins.^28^, ^29^, ^30^ In addition to intracellular targets, some AMPs target nonmembrane extracellular functions. Hepcidin, lactoferrin, haptoglobin, and lipocalin-2 target extracellular iron levels to reduce the iron available for invading pathogens,^31^, ^32^, ^33^, ^34^ whereas indoleamine 2,3-dioxygenase depletes extracellular tryptophan levels to inhibit bacterial growth.^35^ Although it is possible that these mechanisms play a role in the antibacterial action of ANGPT-1, IL-6, and IL-8, they could also potentially contribute to the antibacterial actions of HGF (in addition to membrane permeabilization). The finding that growth factors/cytokines possess antibacterial activity demonstrates, for the first time, their probable dual function in the pulpal response to caries and in the path to dentine-pulp regeneration.

The potential impact of proteinases present in the dental pulp on the antimicrobial factors identified in the current work is worth considering. Previously studies show that peptides with antimicrobial activity are not extensively degraded by enzymes present in dental pulp tissue.^36^ However, larger proteins, such as those described in the current study, are more likely to have multiple sites susceptible to proteolytic degradation. The resultant peptide fragments may or may not retain the antimicrobial properties of the parent molecule.^37^ Therefore, pulpal protease activity could either enhance or diminish the antimicrobial activity of the dental pulp MSC secretome. There is also the possibility that growth factors/cytokines or their degraded peptides could interact synergistically, as has previously been reported for naturally occurring antimicrobial peptides.^38^^,^^39^ Thus, the overall antimicrobial dental pulp MSC secretome is likely to be finely balanced, depending on the levels of individual proteins, their potential for synergistic activity, and their susceptibility to degradation *in vivo*.

It is also worth noting that the culture conditions of the dental pulp MSCs *in vitro* may contribute to the overall abundance of individual growth factors/cytokines, as cells *in vitro* are recognized to adapt to their environment. In the current study, the cell secretome was collected after 24 hours of culture in serum-depleted media, because growth factors present in the serum supplement could have confounded the antibacterial activity results. Serum deprivation has previously been shown to support the survival and replication of MSCs, as well as increasing their angiogenic potential, after 2 to 10 weeks in culture in serum-deprived media.^40^ Although it cannot be ruled out that serum depletion for 24 hours could have contributed to enhancing the levels of angiogenic factors reported herein, it is interesting to note that previous work on dental MSCs from the apical papillae (stem cells from the apical papillae) showed that glucose deprivation and/or hypoxia had more pronounced effects on angiogenic growth factor expression *in vitro* than serum deprivation alone.^41^ In addition, not only culture media but also cell density may influence the content of the MSC secretome. It has been reported that increased cell density of dental pulp MSCs enhances their osteogenic potential^42^ and therefore growth factors/cytokines involved in osteogenesis may be up-regulated under dense culture conditions. Thus, it may be possible to modify the dental pulp MSC secretome *in vitro*, to optimize therapeutic translation for potential tissue regeneration *in vivo*.

To date, improvements in our understanding of the therapeutic potential of dental pulp stem/progenitor have arisen mainly as a result of elegant studies on their regenerative potential.^43^, ^44^, ^45^, ^46^ However, the current work shows, for the first time, that the dental pulp MSC secretome has antibacterial properties and, as such, it has a potential in the resolution of pulpal infection before pulpal regeneration. Any reduction in bacterial growth achieved as a result of bacterial interactions with antimicrobial components of the dental pulp MSC secretome will lower bacteria burden and facilitate further host resolution of infection. The current work therefore provides evidence that, not only odontoblasts^2^^,^^3^ and fibroblasts,^11^^,^^47^ but also other pulp cells, such as dental pulp MSCs, have important antibacterial roles. Thus, following the death of the odontoblast, the underlying pulp tissue should be considered capable of defense, which is an important prerequisite for repair and regeneration.

In conclusion, the current study outlines a novel and diverse antimicrobial secretome from dental pulp MSCs, suggesting that these MSCs harness not only regenerative but also antibacterial properties. With an improved understanding of the secretome of dental pulp MSCs and its novel antimicrobial activity, new evidence for the ability of the dental pulp to fight infection and restore functional competence is emerging, providing further support for the biological basis of pulpal repair and regeneration.

## Supplemental Data

Supplemental Figure S1 Dental pulp mesenchymal stromal cells (MSCs) used in the current study were shown to be positive for the MSC markers CD90 (**A**), CD73 (**B**), CD105 (**C**), and CD44 (**D**) and negative for CD45, CD34, CD11b, CD19, and human leukocyte antigen-DR (all negative markers were contained within a single negative cocktail; **E**); gray peaks represent each fluorophore's respective isotype control. APC, allophycocyanin; FITC, fluorescein isothiocyanate; PE, phycoerythrin; PerCP, peridinin-chlorophyll-protein.

Supplemental Figure S2 Dental pulp mesenchymal stromal cells revealed trilineage differentiation potential toward adipogenic, chondrogenic, and osteogenic lineages *in vitro*. Histochemical staining with Oil Red O showed formation of red lipid droplets after exposure to adipogenic media for 2 weeks (**A**), with Safranin O showed characteristic red/orange staining following exposure to chondrogenic media for 3 weeks (**B**), and with Alizarin Red showed formation of red-stained calcified nodules after culture in osteogenic media for 3 weeks (**C**). Differentiation results were generated using the cells described in Materials and Methods. Differentiation conditions and staining protocols were undertaken as outlined in the study by Pedano et al.^14^ Original magnifications, ×100 (**A**); ×50 (**B** and **C**).
