# Supplementary figures and images for: Novel Antibacterial Properties of the Human Dental Pulp Multipotent Mesenchymal Stromal Cell Secretome
Source: Am J Pathol. 2022 Mar 23;192(6):956–69. doi: 10.1016/j.ajpath.2022.02.005 (PMC12178332; doi:10.1016/j.ajpath.2022.02.005)

A

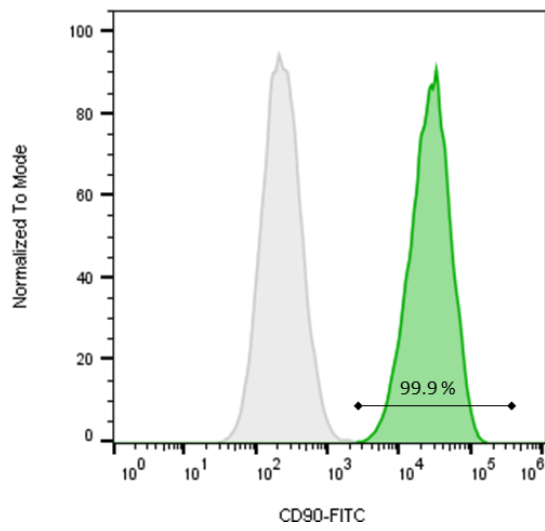

B

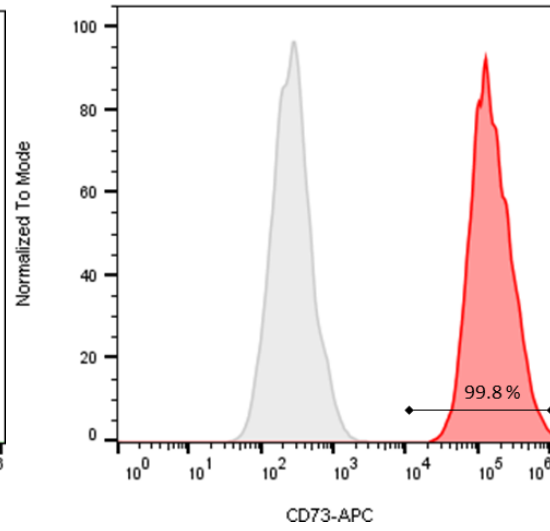

C

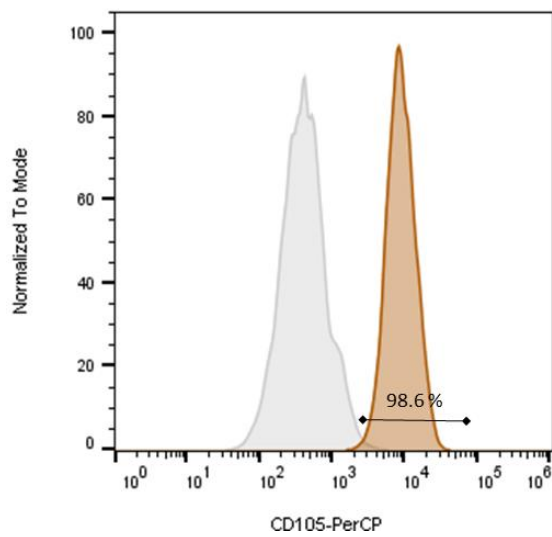

D

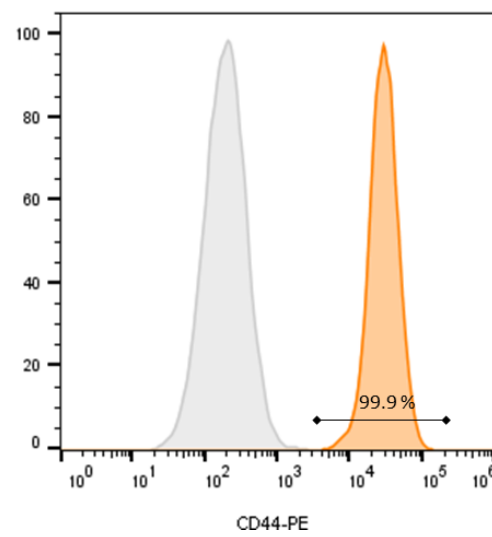

E

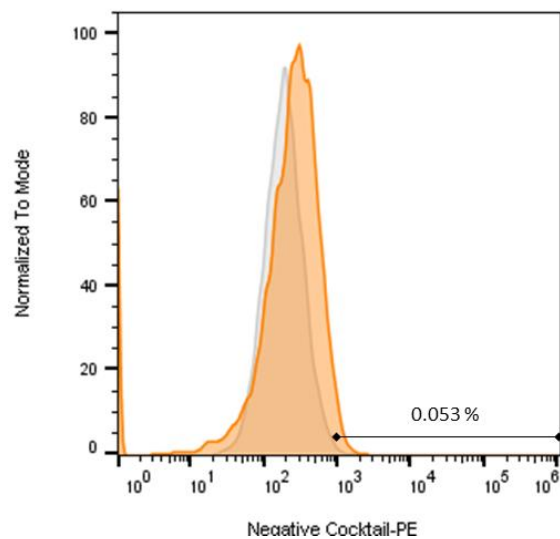

Supplement: Supplemental Figure S1 — Dental pulp mesenchymal stromal cells (MSCs) used in the current study were shown to be positive for the MSC markers CD90 (A), CD73 (B), CD105 (C), and CD44 (D) and negative for CD45, CD34, CD11b, CD19, and human leukocyte antigen-DR (all negative markers were contained within a single negative cocktail; E); gray peaks represent each fluorophore's respective isotype control. APC, allophycocyanin; FITC, fluorescein isothiocyanate; PE, phycoerythrin; PerCP, peridinin-chlorophyll-protein. [file mmc1.pdf]

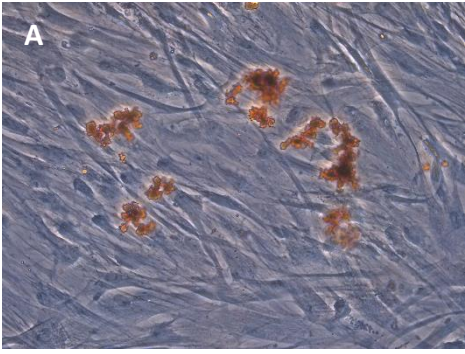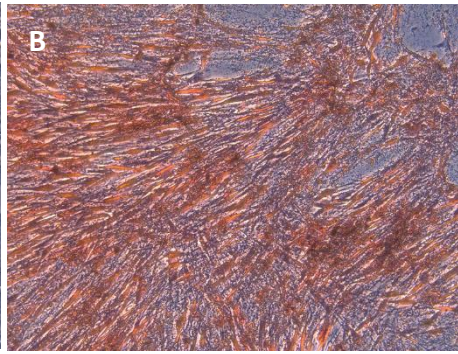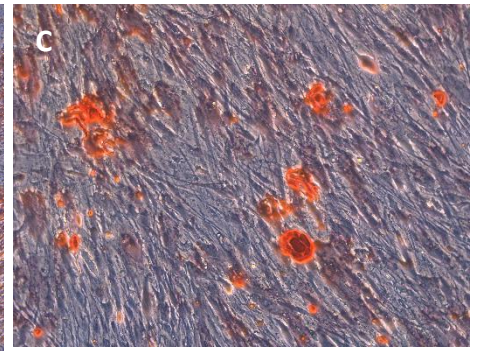

Supplement: Supplemental Figure S2 — Dental pulp mesenchymal stromal cells revealed trilineage differentiation potential toward adipogenic, chondrogenic, and osteogenic lineages in vitro. Histochemical staining with Oil Red O showed formation of red lipid droplets after exposure to adipogenic media for 2 weeks (A), with Safranin O showed characteristic red/orange staining following exposure to chondrogenic media for 3 weeks (B), and with Alizarin Red showed formation of red-stained calcified nodules after culture in osteogenic media for 3 weeks (C). Differentiation results were generated using the cells described in Materials and Methods. Differentiation conditions and staining protocols were undertaken as outlined in the study by Pedano et al.14 Original magnifications, ×100 (A); ×50 (B and C). [file mmc2.pdf]
