# Supplementary material for: Novel Antibacterial Properties of the Human Dental Pulp Multipotent Mesenchymal Stromal Cell Secretome
Source: Am J Pathol. 2022 Mar 23;192(6):956–69. doi: 10.1016/j.ajpath.2022.02.005 (PMC12178332; doi:10.1016/j.ajpath.2022.02.005)
Supplement: Supplemental Table S1 [file mmc3.docx]

Supplementary Table 1. Gene expression profile of AMPs and housekeeping genes in DPSCs. The presence of the AMP lipocalin-2, and lack of AMPs LL-37, β-defensin-2 and β-defensin-3 are demonstrated by qPCR. Average Ct Values of 3 individual experiments performed in duplicate.

| **Gene** | **Average Ct Value** |
| --- | --- |
| *CAMP* (LL-37) | No Ct |
| *DEFB4A* (β-defensin-2) | No Ct |
| *DEFB103* (β-defensin-3) | No Ct |
| *LCN2* (Lipocalin-2) | 38.55 |
| *GusB* (Reference gene) | 26.87 |
| *B2M* (Reference gene) | 20.93 |
